# Exploring the joint potential of inflammation, immunity, and receptor-based biomarkers for evaluating ME/CFS progression

**DOI:** 10.3389/fimmu.2023.1294758

**Published:** 2023-12-20

**Authors:** Uldis Berkis, Simons Svirskis, Angelika Krumina, Sabine Gravelsina, Anda Vilmane, Diana Araja, Zaiga Nora-Krukle, Modra Murovska

**Affiliations:** ^1^ Development and Project Department, Riga Stradins University, Riga, Latvia; ^2^ Institute of Microbiology and Virology, Riga Stradins University, Riga, Latvia; ^3^ Department of Infectology, Riga Stradins University, Riga, Latvia

**Keywords:** Myalgic encephalomyelitis/chronic fatigue syndrome (ME/CFS), immunome, inflammatome, artificial intelligence (AI) supported diagnosis, prognostic and therapy assessment biomarkers

## Abstract

**Background:**

Myalgic encephalomyelitis/chronic fatigue syndrome (ME/CFS) is a debilitating chronic condition with no identified diagnostic biomarkers to date. Its prevalence is as high as 0.89% according to metastudies, with a quarter of patients bed- or home-bound, which presents a serious public health challenge. Investigations into the inflammation–immunity axis is encouraged by links to outbreaks and disease waves. Recently, the research of our group revealed that antibodies to beta2-adrenergic (anti-β2AdR) and muscarinic acetylcholine (anti-M4) receptors demonstrate sensitivity to the progression of ME/CFS. The purpose of this study is to investigate the joint potential of inflammatome—characterized by interferon (IFN)-**γ**, tumor necrosis factor (TNF)-α, interleukin (IL)-2, IL-21, Il-23, IL-6, IL-17A, Activin-B, immunome (IgG1, IgG2, IgG3, IgG4, IgM, and IgA), and receptor-based biomarkers (anti-M3, anti-M4, and anti-β2AdR)—for evaluating ME/CFS progression, and to identify an optimal selection for future validation in prospective clinical studies.

**Methods:**

A dataset was used originating from 188 individuals, namely, 54 healthy controls, 30 patients with a “mild” condition, 73 patients with a “moderate” condition, and 31 patients with a “severe” condition, clinically assessed by Fukuda/CDC 1994 and international consensus criteria. Inflammatome, immunome, and receptor-based biomarkers were determined in blood plasma via ELISA and multiplex methods. Statistical analysis was done via correlation analysis, principal component analysis, linear discriminant analysis, and random forest classification; inter-group differences were tested via nonparametric Kruskal–Wallis *H* test followed by the two-stage linear step-up procedure of Benjamini, Krieger, and Yekutieli, and via Mann–Whitney *U* test.

**Results:**

The association between inflammatome and immunome markers is broader and stronger (coupling) in the severe group. Principal component factoring separates components associated with inflammatome, immunome, and receptor biomarkers. Random forest modeling demonstrates an excellent accuracy of over 90% for splitting healthy/with condition groups, and 45% for splitting healthy/severity groups. Classifiers with the highest potential are anti-β2AdR, anti-M4, IgG4, IL-2, and IL-6.

**Discussion:**

The association between inflammatome and immunome markers is a candidate for controlled clinical study of ME/CFS progression markers that could be used for treatment individualization. Thus, the coupling effects between inflammation and immunity are potentially beneficial for the identification of prognostic factors in the context of ME/CFS progression mechanism studies.

## 1 Introduction

Myalgic encephalomyelitis/chronic fatigue syndrome (ME/CFS) is a debilitating chronic condition for which no diagnostic biomarkers have been identified so far. Its prevalence is at least 0.1% of the population (1), with metastudies reporting values as high as 0.89% (2). In addition, a quarter of patients are bed- or home-bound (3), which highlights the strong need for diagnostic tools as well as biomarkers for the progression and assessment of therapy success. Serum biomarkers are generally used for evaluating disease progression and therapy outcomes; however, validated biomarkers specific to ME/CFS are currently lacking. Recently, several articles have scrutinized broad panels of serum biomarkers in ME/CFS, and with the improved availability of multiplex techniques, simultaneous determination of a wide range of biomarkers from the inflammation–immunity axis has become possible. As a result, clinical practice has now easier access to multimarker panels (4–7), with an emphasis toward inflammatome-, immunome-, or metabolome-associated signatures. The inflammatome–immunome aspect in ME/CFS has been reviewed in (8). In practice, biomarker panels will require large cohorts to detect and validate the ME/CFS-specific domain in the multidimensional vector space for direct application of the test values; therefore, aggregate evaluations can be preferable, at least, from a practicability viewpoint. Since one of the ME/CFS traits is that individual inflammation–immunity biomarker values remain within the normal range, a difference from healthy controls in the multidimensional space will not necessarily be of sufficient decisive power. Our study addresses this challenge by proposing an aggregate type of biomarker analysis.

Recently, several serum biomarkers have been proposed as sensitive indicators for the severity of the condition: Activin-B (9), creatine kinase (CK), and creatinine (5, 10), as well as anti-β2AdR and anti-M4 (11). Lower CK levels, which suggest muscular underuse, have been observed in the severe condition group (10) where prolonged physical inactivity correlates with symptom severity. In turn, CK levels could be a correlate of physical inactivity. It should be noted, however, that the interpretation of lower serum levels of intracellular enzymes will require complex homeostatic models, which are not yet available. Even though there is increased interest in the homeostatic mechanisms underlying the ME/CFS condition (12, 13), the explanatory models are not yet equipped with behavioral variables like physical activity and physiological determinants such as the motion range in an ambient setup. It must be noted that earlier reports on similar postviral fatigue syndrome have found elevated CK levels (14); therefore, precision-medicine-based ME/CFS cohort studies will be necessary to establish the diagnostic toolkit of reduced CK levels. While these serum biomarkers can be useful for therapy assessment, there is also an interest in biomarker-based assessments that are not solely reliant on the lower bound of intracellular enzymes, which can be challenging to establish.

Therefore, a different type of multimarker panel, where the link to a particular symptom—as the case of reduced muscle use and CK decrease—is not necessarily direct, is highly relevant. Combined criteria applicable even when individual serum biomarkers are in the normal range are essential, and for use in clinical practice, they must be integrated into an artificial intelligence (AI)-based decision support system. In particular, biomarkers for the management of mild and severe condition remain an unmet need; otherwise, panels sensitive only to the entry into the severe condition would be used for detection in the healthcare system. Diverse types of biomarker-based assessments would be preferred for combination in AI systems, with a data fusion perspective between different types and modalities of clinical and healthcare data having the purpose of severity classification.

The broader availability of multiplex methods allows a rapid determination of multimarker panel values. However, these methods also have limitations: enveloping the subsets of patients with different degrees of ME/CFS severity in the multidimensional vector space will require large cohorts for validation. Therefore, aggregate indicators or AI-based algorithms would be preferred for ME/CFS multimarker validation and implementation. AI is an important enabler for healthcare practitioners, to use diagnostic algorithms of high complexity.

Studies focusing on ME/CFS natural history and addressing the transition from different severity states to recovery are rare. One study on the subject (15) analyzes its theoretical aspect, whereas others (16–18) address the question of reversibility and recovery. Studies show that the basal recovery rate in ME/CFS is rather low (19). In this context, our approach allows discussing the condition reversibility based on biomarker panel use, thus complementing the symptom-based reversibility measurements. There have been several clinical trials for ME/CFS with various success rates (20), with some falling into the domain of the inflammation–immunity approach. Remarkably, very few studies address the dynamic observation of patients with ME/CFS, with information on biomarker levels obtained at fixed intervals. Therefore, conclusions from population-based studies with determination of different stages of ME/CFS are currently projected to the potential clinical course of a ME/CFS patient. Remarkably, patients with a substantial improvement in their status are prone to lose contact with the healthcare system, which does not fully address their expectations (21), thus becoming less available for studies.

Studies on outbreaks (22) and infection waves constitute a strong opportunity for hints concerning external causative factors. In contrast, in the absence of mechanistic models and consent biomarkers, statistical interpretation remains largely intuitive. For example, regarding the COVID-19 pandemic as a trigger for ME/CFS, while the overall picture remains blurred (23), studies observe the interrelation between long-COVID and ME/CFS (24) on macroscopic symptoms as well on a biomarker level. ME/CFS is gaining additional interest following the outcomes of the COVID-19 pandemic, emergence of long-COVID, and the overall increase in the chronic disease burden (25).

While there have been more extensive findings for biomarkers for the severe condition group [(5) mentions CK, creatinine], the lack of biomarker signature for moderate and mild conditions limits the application of therapeutic approaches before the onset of the severe stage. Novel research on cell-based biomarkers has the potential to detect earlier stages of ME/CFS (26); however, methods involving high specificity also imply complex technical solutions (27) and are not yet implemented in clinical practice. Biosensors have the potential for clinical validation and monitoring in ME/CFS.

Our study aims to investigate the potential of inflammatome—characterized by interferon (IFN)-**γ**, tumor necrosis factor (TNF)-α, interleukin (IL)-2, IL-21, Il-23, IL-6, IL-17A, Activin-B, immunome (IgG1, IgG2, IgG3, IgG4, IgM, and IgA), and highlighting autoantibody type receptor-based biomarkers (anti-M3, anti-M4, and anti-β2AdR)—for evaluating condition progression in patients with ME/CFS and identifying the optimal selection to assess prospective clinical tests for ME/CFS. Anti-M3, anti-M4, and anti-β2AdR as receptor-based biomarkers are analyzed separately from immunome as there is recently highlighted interest in their diagnostic and explanatory role (11, 28).

## 2 Materials and methods

### 2.1 Patients

An observational study of a cross-section of ME/CFS severity groups has been carried out between May 2020 and December 2022. The Fukuda/CDC-1994/case definition criteria (29, 30) were used to determine the initial clinical diagnosis with physicians’ clinical evaluation of inclusionary symptoms and exclusionary illnesses in accordance with the clinical protocol. Before inclusion in the study, the diagnosis was verified according to the International Consensus Criteria (31). The condition severity has been assessed by the visual analog scale (VAS, ranging from 0 to 10) for pain, the Athens Insomnia Scale (32), and an adapted semi-structured survey (33) consisting of 27 questions structured in six sections: causes and triggers of fatigue; character of fatigue; current symptoms; comorbidities; solutions for fatigue; and its influence on work disability and allowing multiple choice answers. Patients were managed and health records were stored at the Rīga Stradiņš University (RSU) Ambulance outpatient clinic. Blood samples were collected by a certified nurse. A total of 54 blood samples from healthy donors from the Latvia State Blood Donor Center were included in the study as a control group. The characteristics of the study groups are described in **Table 1**.

**Table 1:** Study group characteristics.

	Healthy controls (0)	Mild (1)	Moderate (2)	Severe (3)	*p*-value
Number of cases	54	30	73	31	
Sex Female/Male	13/41	24/6	47/26	21/10	*p*_χ_^2^ < 0.001 0 vs. 1 0 vs. 2 0 vs. 3
Age median (IQR)	26 (20–33)	55 (42–64)	50 (38–61)	39 (34–53)	*p*_KW_ < 0.001 0 vs. 1 0 vs. 2 0 vs. 3 *p* < 0.05 1 vs. 2 1 vs. 3

### 2.2 Laboratory analysis

Plasma samples of patients and healthy controls were analyzed for autoantibodies against muscarinic cholinergic receptors 3 (M3) and 4 (M4) using ELISAs according to the manufacturer protocols from CellTrend, GmbH (Luckenwalde, Germany). Samples from patients with ME/CFS were analyzed for anti-β2AdR antibodies using the validated human β2AdR surface quantitative ELISA kit (BlueGene, Shanghai, China). Human plasma Activin-B concentration was analyzed using the commercially available ELISA kit (LifeSpan BioSciences, Seattle, WA, USA).

Detection of inflammatome members IL-2, IL-17, IL-6, IL-21, IL-23, TNF-α, and IFN-γ in plasma samples of patients with ME/CFS and healthy controls was carried out with a Luminex 200 instrument system using a commercially available kit (MILLIPLEX MAP Human high sensitivity T cell panel-immunology multiplex assay, Merck KGaA, Darmstadt, Germany) according to the manufacturer’s protocol.

Detection of immunome members IgM, IgG1, IgG2, IgG3, IgG4, and IgA in plasma samples of patients with ME/CFS and healthy controls was carried out with Luminex 200 instrument system using a commercially available kit (MILLIPLEX MAP Human Immunoglobulin Isotyping Magnetic Bead Panel, Merck KGaA, Darmstadt, Germany) according to the manufacturer’s protocol.

### 2.3 Statistics

Data analyses and graphs were performed with GraphPad Prism 9.0 for MacOS software (GraphPad Software, San Diego, CA, USA), JMP 16 (SAS, Cary, NC, USA), and JASP 0.18 (34). Statistical significance was set at *p* < 0.05.

The issue of missing values in the analyzed dataset was managed with JMP Pro 17.0 using multivariate Robust Principal Component Analysis (RPCA) imputation algorithm, which replaces missing values using a low-rank matrix factorization with singular value decomposition (SVD) as a postcompression procedure. Statistical modeling was performed using JMP, JASP, and R. Principal component factoring was used to identify the underlying structure of the data, with scree plot compared with values from parallel analysis used for determination of the number of components.

Normality of numerical data was determined using the D’Agostino and Pearson and Shapiro–Wilk normality tests, homogeneity of variances was checked by Brown–Forsythe and Bartlett’s tests, and since a predominantly logarithmic distribution of the data was observed, comparative concentrations of serum biomarkers in healthy controls and patients with varying degrees of ME/CFS severity were analyzed using the nonparametric Mann–Whitney (MW) *U*-test or Kruskal–Wallis (KW) *H*-test with Benjamini, Krieger, and Yekutieli’s two-stage step-up procedure as a *post-hoc* test. To characterize the magnitude of differences found, the effect size of variable pairs was determined using Cohen’s *d* for Student’s *t*-test and matched rank biserial correlation for Wilcoxon signed-rank test (35).

In the case of the Gaussian distribution, a nomogram (36) was used to calculate sample size and power for normally distributed variables (36). The median with IQR was used to characterize the central tendency and dispersion of the variables. The numbers represent exact *p*-values and *p*-values <0.05 were assumed to be significant.

The direction and strength of associations between studied variables in healthy controls and patients with ME/CFS with different condition severity were analyzed using Spearman’s ρ; correlation network plots of associations between studied variables in healthy controls and patients with ME/CFS with different condition severity were also created. To compare the proportions of respective cases in a categorical classification between the groups, the Chi-square (χ^2^) test was applied.

The random forest analysis for classification problems to severity classes based on combined inflammatome–immunome and for a separation of healthy controls from patients with ME/CFS was performed, and a decision tree plot was created. Number of trees was set at 5,000: using the test set allows avoiding the overfitting problem; in addition, this allows a coherent comparison with the outcomes in (28) for a similar composition of variables assessed in both studies. Otherwise, we have used default/internally optimized values via the calculation package.

### 2.4 Ethics approval

The study design was approved by the Ethical Committee of Rīga Stradiņš University (code Nr.6-1/05/33; date of approval: 30 April 2020) and written consent was obtained from all participants.

## 3 Results

### 3.1 Differences between ME/CFS and healthy controls

**Figure 1:**
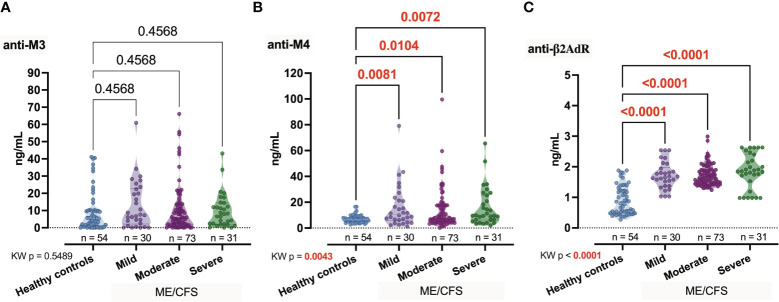
Comparative concentration of studied autoantibodies anti-M3, anti-M4, and anti-β2AdR **(A–C)** in healthy controls and patients with ME/CFS depending on condition severity. Numbers represent exact *p*-values [Kruskal–Wallis (KW) with two-stage linear step-up procedure of Benjamini, Krieger, and Yekutieli as *post-hoc* test]. KW value represents the overall test outcome, whereas the *post-hoc* intergroup comparisons are shown above, with statistically significant differences represented in red.

Among the candidate autoantibodies, anti-M4 showed significant (*p*_KW_ = 0.0043, *H* = 13.17) differences between the healthy controls and patients with different severity degrees of ME/CFS [Healthy controls (Ctrl) vs. Mild, *p* = 0.0081; Ctrl vs. Moderate, *p* = 0.0104; Ctrl vs. Severe, *p* = 0.0072; **Figure 1B**]. However, the anti-M4 intragroup median does not monotonically increase with increasing severity of the disease. Compared to the control group, the increase in the level of anti-β2AdR was also statistically significant, but more pronounced (*p*_KW_ < 0.0001, *H* = 74.69; **Figure 1C**) and for all intergroup comparison with Ctrl *p* < 0.0001, that is in line with our earlier research (11) and findings of other authors (28). Finally, anti-M3 did not show differences between the groups (*p*_KW_ = 0.5489, *H* = 2.12; **Figure 1A**).

Significant intergroup differences have also been observed for IL-2 (*p*_KW_ = 0.0005, *H* = 17.66), IL-21, (*p*_KW_ = 0.0005, *H* = 17.56), and IL-6 (*p*_KW_ = 0.0026, *H* = 14.26) (**Figure 4**). For IL-17A (*p*_KW_ = 0.0320, *H* = 8.81; **Figure 4G**) and Activin-B (*p*_KW_ = 0.0151, *H* = 9.86; **Figure 4H**), only some of the differences were significant (IL-17A, Ctrl vs. Moderate, *p* = 0.0255; Activin B, Ctrl vs. Moderate, *p* = 0.0160, Ctrl vs. Severe, *p* = 0.0160).

**Figure 2:**
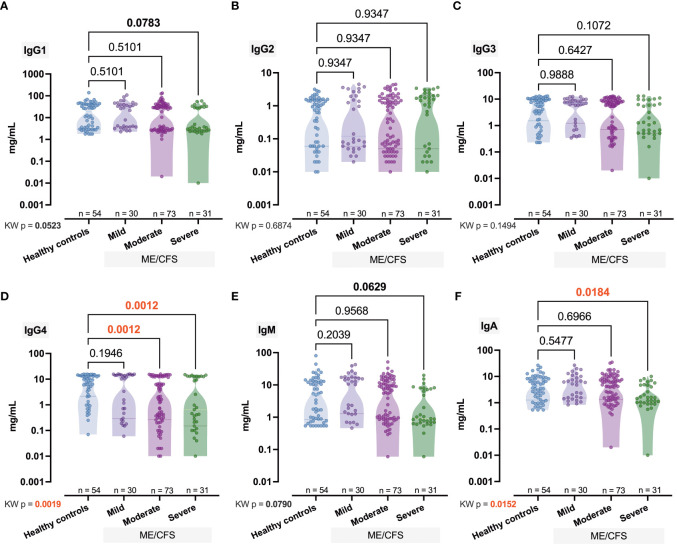
Comparative concentration of immunome represented via immunoglobulins IgG1, IgG2, IgG3, IgG4, IgM, and IgA **(A–F)** in healthy controls and patients with ME/CFS with different condition severity. Numbers represent exact *p*-values [Kruskal–Wallis (KW) test with two-stage linear step-up procedure of Benjamini, Krieger, and Yekutieli as *post-hoc* test]. KW value represents the overall test outcome, whereas the *post-hoc* intergroup comparisons are shown above, with statistically significant differences represented in red.

Next, for the immune biomarkers we found that IgG4 levels decrease with increasing severity — medians for Mild, Moderate, and Severe groups were 10.24, 1.63, and 0.65 mg/ml respectively (*p*_KW_ = 0.0019, *H* = 14.90; **Figure 2D**). A less pronounced (*p*_KW_ = 0.0152, *H* = 10.43) effect was observed in the case of IgA, and only in the Severe group was the IgA median level significantly (*p* = 0.0184) lower than Ctrl (1.55 vs. 4.02; **Figure 2F**). Our study revealed that the level of IgM increased in comparison to control (5.1) toward the mild (11.59) and moderate (5.86) stages but significantly fell sharply (0.91) in the severe stage (*p*_MW_ = 0.0283; **Figure 2E**). A similar effect was observed in the case of IgG3 [Ctrl (6.15) vs. Severe (1.28), *p*_MW_ = 0.0319; **Figure 2C**] and IgG1 [Ctrl (19.33) vs. Severe (3.30), *p*_MW_ = 0.0274; **Figure 2A**]. The respective median values of the IgG2 level in Ctrl, Mild, Moderate, and Severe groups were 0.84, 0.12, 0.40, and 1.44 mg/ml (**Figure 2B**).

### 3.2 Analysis of associations

**Figure 3:**
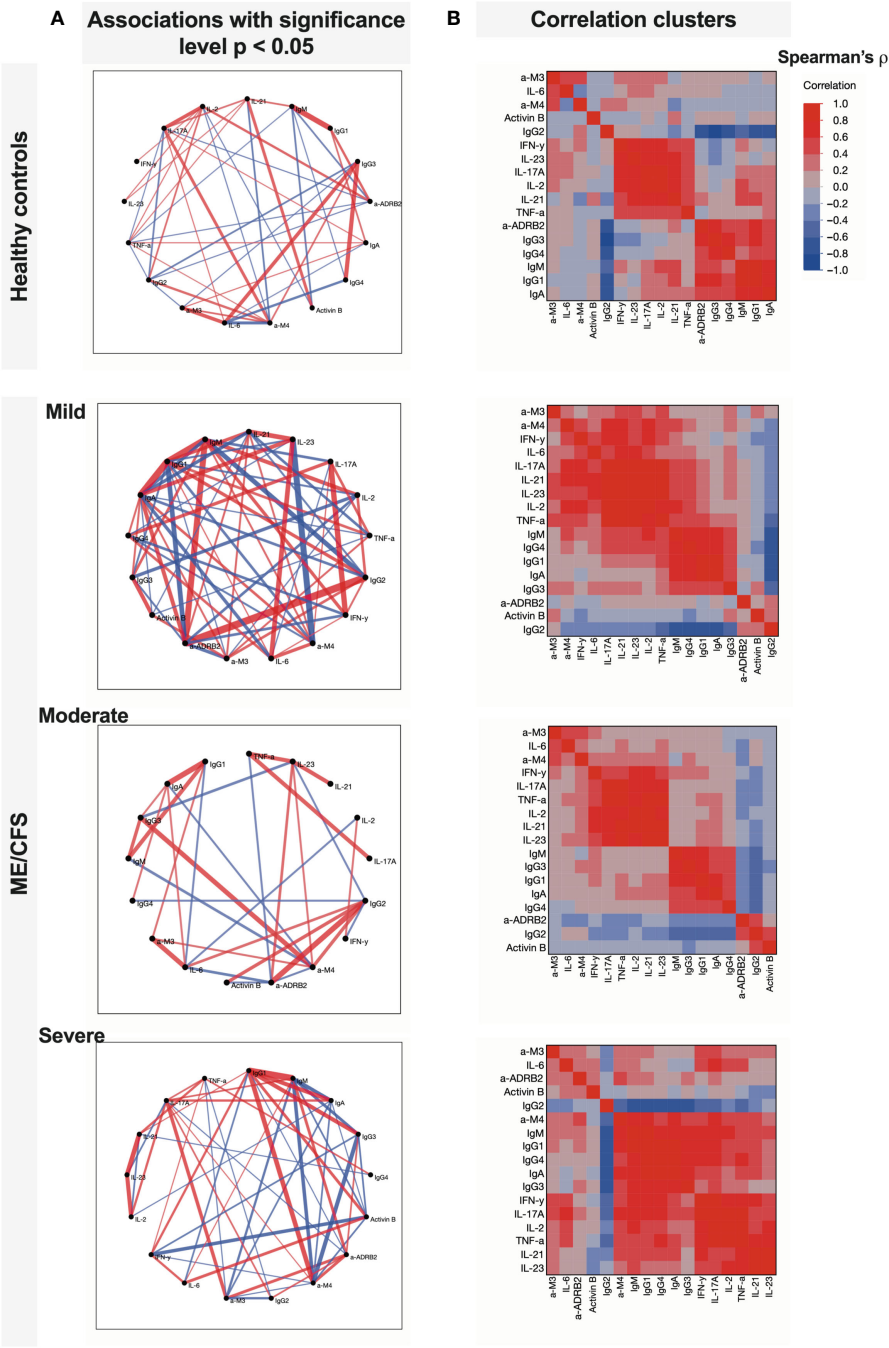
Comparison of associations between studied variables in healthy controls and patients with ME/CFS with different condition severity. **(A)** Partial correlation diagrams showing direction and strength of significant associations (reflecting Spearman’s ρ with *p* < 0.05); **(B)** correlation clusters that show grouped variables with similar interactions.

According to the correlogram in **Figure 3B**, in the severe condition group, a large cluster is established corresponding to the coupled appearance of immunome- and inflammatome-related biomarkers. A large number of links in **Figure 3A** corresponding to mild condition reflects the initial state when the condition starts to establish (triggering). However, two clusters are still recognized, namely, healthy controls and the moderate state of the condition. In the severe ME/CFS group, those subclusters fuse to form a large cluster of positive-feedback biomarkers, and the interaction of inflammatome with immunome is observed: a correlated fused cluster of variables IFN-γ, IL-17A, IL-2, IL-21, IL-23, TNF-α, anti-M4, IgM, IgG1, IgG3, IgG4, and IgA. Remarkably, for healthy controls, inflammatome and immunome demonstrate well-separated clusters of correlation. Correlation analysis of healthy controls vs. patients with ME/CFS (without extraction of severity group) demonstrates the changes in the network of significant associations (**Figure 5**). For healthy controls, the associations are centered around immunome and inflammatome with a limited number of interactions. In the ME/CFS group, there is a dense network of crosstalk between the immunome and inflammatome. Activin-B is not significantly linked to either cluster in healthy controls, whereas significant links emerge for the condition group. Thus, Activin-B could indicate involvement in the triggering mechanism of ME/CFS.

**Figure 4:**
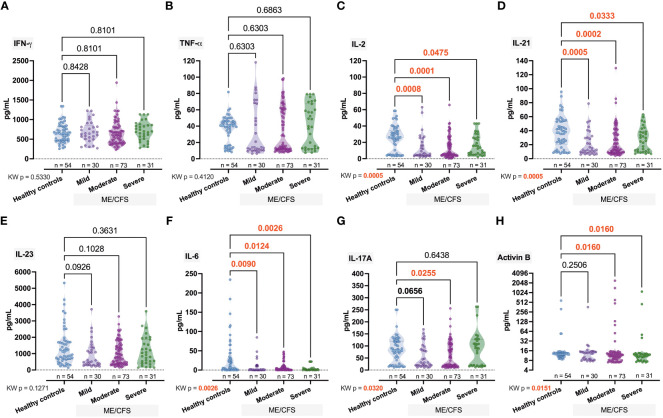
Comparative intergroup concentration of inflammatome cytokines interferon (IFN)-γ, tumor necrosis (TNF)-α, interleukin (IL)-2, IL-21, IL23, IL-6, IL-17A, and Activin-B **(A–H)** in healthy controls and patients with ME/CFS with different condition severity. Numbers represent exact *p*-values [Kruskal–Wallis (KW) with two-stage linear step-up procedure of Benjamini, Krieger, and Yekutieli as *post-hoc* test]. KW value represents the overall test outcome, whereas the *post-hoc* intergroup comparisons are shown above, with statistically significant differences represented in red.

**Figure 5:**
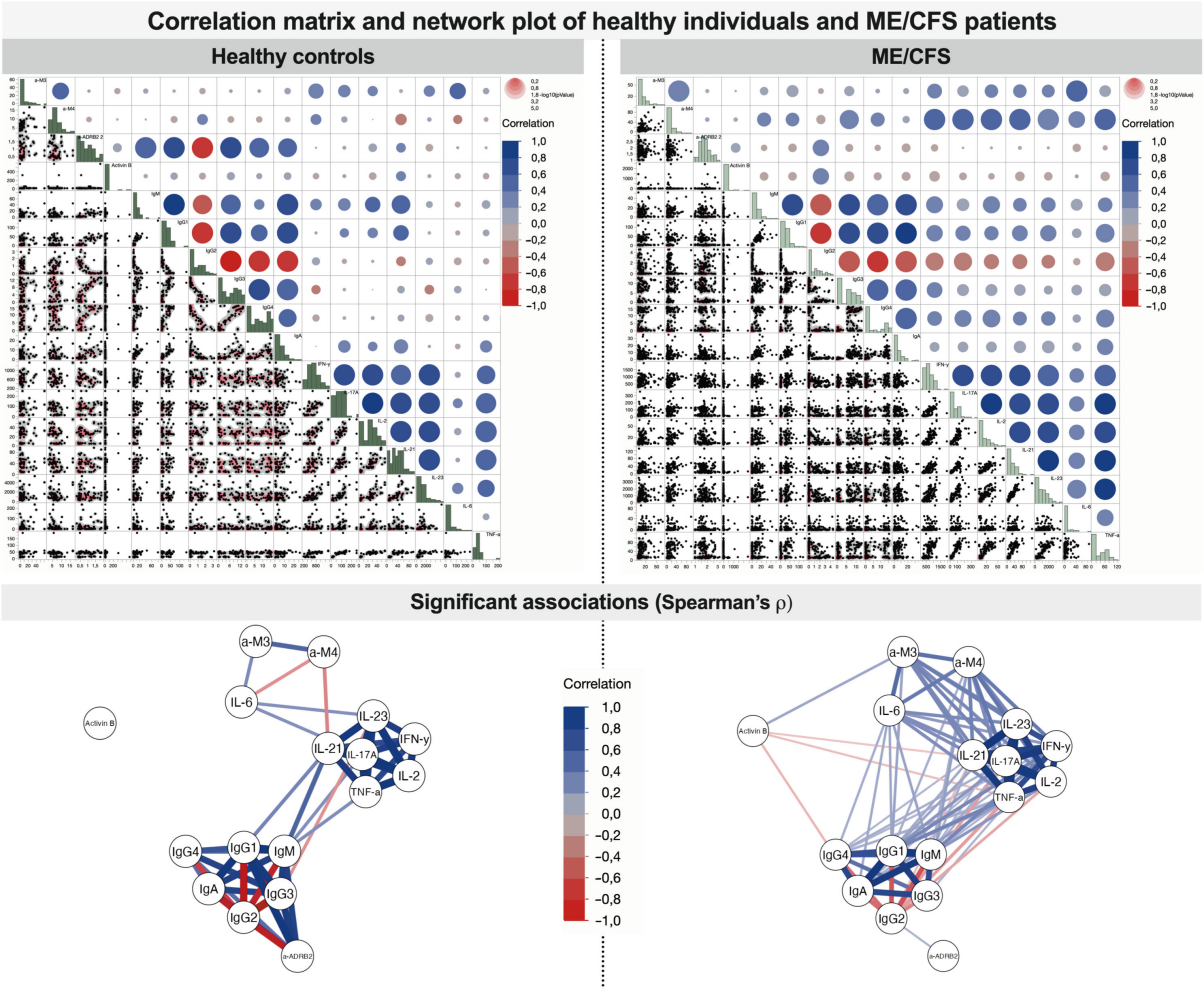
Correlation matrix and network plot of associations between studied variables in healthy controls and patients with ME/CFS (all severity groups joined). Significant associations reflect Spearman’s ρ significance test with *p* < 0.05.

**Figure 6:**
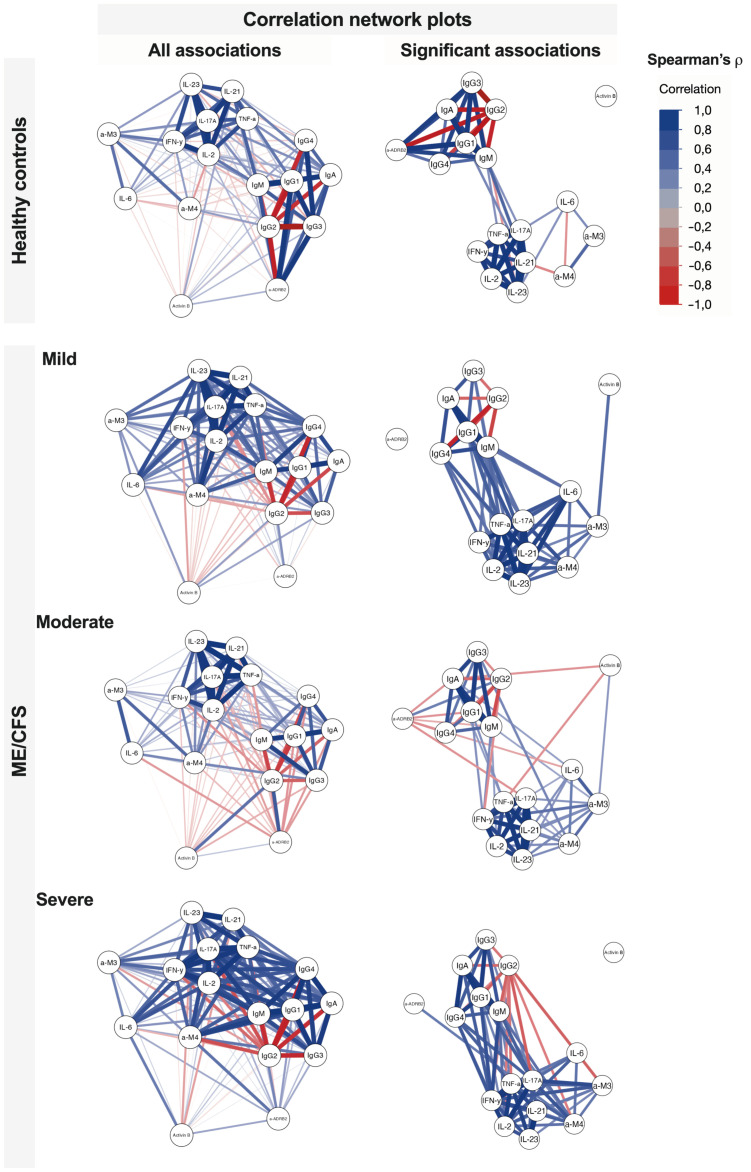
Correlation network plots of associations between studied variables in healthy controls and patients with ME/CFS with regard to different condition severity. Significant associations reflect Spearman’s ρ with *p* < 0.05.

Correlation networks more profoundly demonstrate the relative separation of immunome and inflammatome for healthy controls and earlier stages of ME/CFS and their coupling for the severe condition group (**Figure 6**). Activin-B acts as a candidate for a trigger-associated biomarker at the beginning of the condition, then bridging immunome and inflammatome, and exiting the game for the severe established condition.

### 3.3 Linear discriminant classification

**Figure 7:**
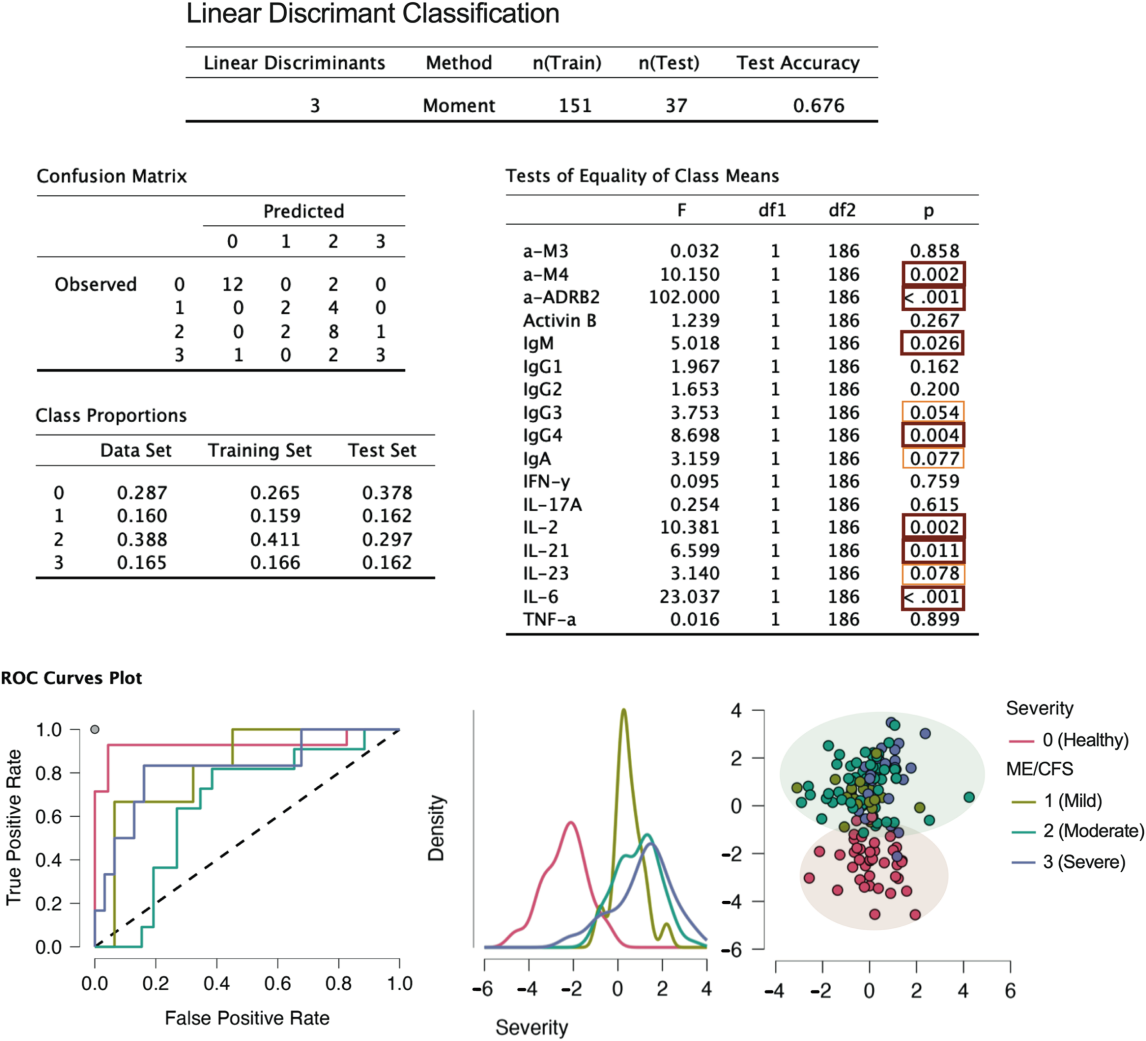
Linear discriminant classification analysis.

Linear discriminant classification (37) was employed to assess the separation between healthy controls and ME/CFS cases, as well as among different severity groups (**Figure 7**). While the discrimination between controls and cases was highly efficient, the distinction of the different severity groups via the linear method was not achieved, as shown by the two separated bubbles. Therefore, additional methods will be employed in the data analysis to better capture the structure of severity classes.

### 3.4 Principal component analysis

**Figure 8:**
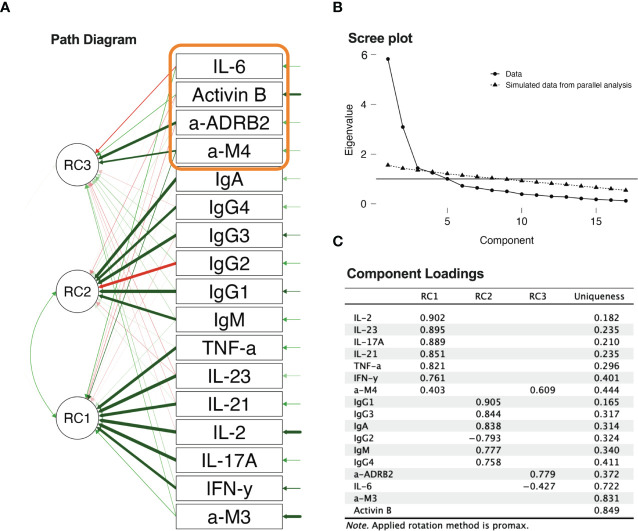
Principal component analysis: **(A)** path diagram of principal component factoring; **(B)** scree plot of component number determination; **(C)** component loadings from principal component factoring.

Exploratory principal component factoring was conducted to identify the underlying structure of the biomarker data (**Figure 8**). Based on the scree plot and parallel analysis, a number of components for further factor rotation have been selected (38) On the scree plot, the eigenvalue where the line becomes horizontal is the third one. The corresponding eigenvalue is also slightly over the simulated eigenvalue from the parallel analysis (38, 39). Principal component factoring over data containing all groups rotated to separation between inflammatome, immunome, and receptor biomarkers. The promax oblique method was used for rotation, as oblique conformation was expected there, due to the interdependence of inflammation and immunity-dependent processes and resources. The first factor (RC1) was associated with the inflammatome, the second factor (RC2) was associated with the immunome (specifically Ig classes), and the third factor was characterized by anti-M4 and anti-β2AdR biomarkers, which have been previously shown to be significant in group distinctions. Anti-M4 played a bridging role between the inflammatome and immunome. The presence of negative IgG2 and IL-6 coefficients can be associated with exhaustion and depletion [see, for instance, (40)]. Finally, the high uniqueness of Activin-B suggests that it can be attributed to its association with early events after ME/CFS onset.

### 3.5 Random forest classification

**Figure 9:**
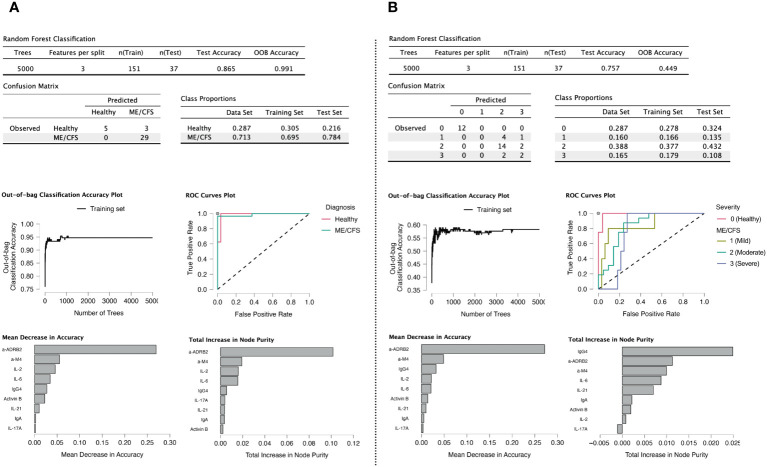
Random forest modeling (41). **(A)** Classification of healthy controls (HC) vs. cases. **(B)** Classification into HC and severity groups. Variables having negative values for decrease in accuracy were eliminated from the model, as it means their presence is counterproductive.

Variable importance plots reveal that anti-β2AdR is the most relevant marker for accuracy and purity in both healthy controls and patients with ME/CFS (**Figures 9A, B**), consistent with our previous research (11). Anti-M4 also shows a high score on both plots. The high scores of IgG4 and IL-2 may indicate the presence of an autoimmune process underlying the ME/CFS onset. In addition, the relatively high score of Activin-B is in line with the findings from the principal component factoring analysis. The plot of number of trees vs. OOB accuracy indicates that the oscillation of OOB no longer occurs at the selected tree number.

## 4 Discussion

Intergroup comparison of inflammation biomarkers in our study reveals several statistically significant differences. These results were compared with a previous study (4) that relied on ME/CFS duration since onset as a continuous severity measure and regression of cytokine levels on severity as the analysis method, and another one (7) where severity was projected to short- and long-term condition duration and *t*-test was used. In comparison, IL-2 and IL-6 demonstrated an increasing tendency in the study (4) although not significant. In contrast, IL-6 shows significant differences in the study (7), with the highest levels in the short disease duration group. IL-17F from the IL-17 family measured in (4) shows a significant difference, while IL-17A shows a strong significance in (7). IL-21, IL-23, and Activin-B were not measured. TNF-α difference was not significant in (4) but was significant in (7). The only case where the upward trend is significant in both studies, although the KW test in our study is not (**Figure 4A**), will be IFN-γ; remarkably, in a different study (40), IFN-γ shows no intergroup significant difference. Activin-B has produced varying results in earlier studies (9, 42).

For immune biomarkers, in a previous study (43), IgG3 or IgG4 deficiency has been described in ME/CFS, which corresponds to our finding for IgG4. Additionally, this study reports the elevation of IgM and IgG2 levels, which are not statistically significant in our study.

A positively correlated inflammatome–immunome cluster for the severe ailment group is indicative of a possible positive feedback mechanism that is yet unknown. This feedback is consistent with the hypothesis of a homeostatic suboptimal state when the condition has developed in a protracted time span after the initial triggering event. It also underpins the observed aggravated symptoms caused by inflammation, where fatigue fits in and is observed well in studies on immune and rheumatic diseases. It can be hypothesized that in the mild and moderate states, the not yet established feedback allows for keeping substantial autonomous functioning capabilities for the patient. Activin-B serves as an indicator of an evolving condition.

The causality underlying the research is of utmost importance for basic research, and correlation alone will not answer this challenge. However, for the progression and recovery monitoring, any pathological-process-related biomarker is of great importance, as is the case for the positive feedback signature. To quantify this effect, we can require four or more inflammatome elements to have a substantial correlation with four or more elements of the immunome. Such an index could be prospectively validated in ongoing ME/CFS studies.

The separation between the inflammatome and immunome in principal component factoring strengthens the assumption about different partial mechanisms underlying the progression of ME/CFS. If this is one process, it must be substantially non-linear, triggering and cascading various stages of the condition. Understanding of the potential circuit of interaction would facilitate the development of therapies able to break the unfavorable circuit. Separation of components justifies analysis of corresponding correlation patterns among severity groups.

The multi-marker environment with complex interlinks requires an AI-based toolkit for decision support. The resulting outcomes when stacking various AI-based decision machines have yet to be determined in the case of ME/CFS [see (44) for a novel stacking approach]. Clearly, the inflammatome–immunome interaction is a criterion that complements machine-learning-based AI systems as described in (45). Coherence with Activin-B having high variable importance indicates robust variable importance in an intergroup AI-based classification (9, 45). Our study uses a comparatively large dataset, thus contributing to robust and stable variable selection resulting from machine learning.

However, the lack of studies on ME/CFS pathogenetic dynamics severely limits widening the AI solutions with patient-centered measures, as opposed to studying cross-sectional snapshots from populations usually at the primary diagnostic event. At the same time, dynamical observation of patients is not burdensome, and biomarker measurements can be performed at regular intervals. Thus, the availability of prognostic factors that are able to predict disease progression will facilitate the use of therapies and allow the timely monitoring of the outcomes of therapeutic approaches.

The differences between biomarker validation results observed in various studies for progression biomarkers are affected by different case definitions as well as different outcomes to be measured. Whereas a self-reported severity measure is commonly used, other quantifiable biomedical assessment methods have been proposed (9, 46). Furthermore, a controlled clinical trial would be preferable for diagnostic and progression biomarkers in order to standardize inclusion/exclusion and assessment methods; however, this can be complicated since the endpoint is not easily associated with a measurable biomarker for ME/CFS. Here, the solution could be a personal digital assistant capturing the information related to severity stage transition, probably paired with a pertinent biosensor and wearables.

If validated in further studies, the inflammatome–immunome interaction will further justify ME/CFS as a systemic complex disease following a triggering event, with inflammation and immune response aberration, instead of defining it as a mood disorder or mental ailment.

In random forest analysis, the highest placement of anti-β2AdR is consistent with statistical analysis, as is also the case for anti-M4. The appearance of IgG4 on the highest position of node purity for severity group classification indicates that receptor-indicated processes are associated with immunome-based indicators for a longer-lasting condition.

The strength of the current study is a relatively large dataset obtained during the investigation, and also the care organization in Latvia, where the RSU outpatient clinic is a single specialized center for ME/CFS. This secures representativity of cases for ME/CFS in the population. The study is based on primary data, obtained for the study by the same staff and using the same certified laboratory equipment for respective measurements. One limitation of the study is that the study outcome is still a severity group, and not a continuous severity index, which could make more informative regression models attainable. With the advent of biosensors, activity trackers and digital assistant collection of objective continuous data become achievable. The second half of the study period started to overlap with the first arrival of long-COVID cases, but we remained very strict with our inclusion/exclusion criteria. However, future ME/CFS studies will be greatly affected by the impact of long-COVID appearance, which may be advantageous, but also disadvantageous in an environment of strong informative noise. We did not include pediatric cases, nor were there cases in Latvia available with a very (extremely) severe ME/CFS. Furthermore, our study does not cover a complete set of inflammation–immunity-related biomarkers, e.g., the IL-10 family is not included; nevertheless, the conclusions are not affected by the non-inclusion of additional biomarker sets.

## 5 Conclusions

Mid- and long-term observation of ME/CFS courses is needed to reveal relationships about the transition between severity groups and to associate them with relevant biomarkers. A participatory design is required because the varying incidence of clinical symptoms, combined with AI, will be valuable for progress and therapy evaluation. A meaningful combination with biomarker measurements and with increasing datasets finally has the perspective of forming a basis for clinical trials for diagnosis and therapy. ME/CFS research needs longer tailored projects compared to the usual 3-year duration practice prevailing in Europe to create representable datasets about condition dynamics in cohorts.

Our study reveals a specific correlative relationship between collections of inflammatory, immune, and receptor-based biomarkers unequivocally characterizing the severe condition group. Further investigations via principal component analysis and random forest classification underpin the observed correlation as an established feature of ME/CFS, with a perspective to develop a diagnostic test in a clinical trial.

The immunome–inflammatome association as a diagnostic test would make sense also from a technology assessment viewpoint. The broader availability of multiplex systems makes multimarker tests more available in routine laboratory services. The highest classification power from data analysis is attributed to anti-β2AdR, anti-M4, and IgG4, as well as IL-2 (an autoimmune processes marker) and IL-6—the marker for innate immunity. From the viewpoint of monotonicity, a composite index will be needed to reflect the disease “progression”, as anti-β2AdR and anti-M4 show an up and down behavior among severity groups.

The progression measurement supported by an aggregate marker will be relevant for integration into the healthcare system—family doctors/GPs require the availability of simple diagnostic algorithms and AI tools with few parameters. Combining the classical biomarker panel method with aggregate progression assessment via a correlation matrix provides a new approach to ME/CFS diagnostics.

## Data availability statement

The raw data supporting the conclusions of this article will be made available by the authors, without undue reservation.

## Ethics statement

The studies involving humans were approved by Ethical Committee of Rīga Stradiņš University (code Nr.6-1/05/33 and date of approval 30 April 2020). The studies were conducted in accordance with the local legislation and institutional requirements. The participants provided their written informed consent to participate in this study.

## Author contributions

UB: Conceptualization, Formal analysis, Investigation, Writing – original draft, Methodology. SS: Data curation, Software, Writing – original draft, Conceptualization, Investigation, Methodology, Resources, Visualization. AK: Methodology, Writing – original draft, Investigation, Resources. SG: Investigation, Writing – review & editing. AV: Data curation, Investigation, Writing – review & editing. DA: Data curation, Resources, Writing – review & editing. ZN-K: Validation, Writing – review & editing. MM: Conceptualization, Funding acquisition, Project administration, Resources, Validation, Writing – review & editing, Supervision.

## References

[1] Estévez-López F Mudie K Wang-Steverding X Bakken IJ Ivanovs A Castro-Marrero J. Systematic review of the epidemiological burden of Myalgic Encephalomyelitis/Chronic Fatigue Syndrome across Europe: Current evidence and EUROMENE research recommendations for epidemiology. J Clin Med (2020) 9(5):1557. doi: 10.3390/jcm9051557 32455633 PMC7290765

[2] Lim E-J Ahn Y-C Jang E-S Lee S-W Lee S-H Son C-G. Systematic review and meta-analysis of the prevalence of chronic fatigue syndrome/myalgic encephalomyelitis (CFS/ME). J Transl Med (2020) 18(1):100. doi: 10.1186/s12967-020-02269-0 32093722 PMC7038594

[3] Pendergrast T Brown A Sunnquist M Jantke R Newton JL Strand EB. Housebound versus nonhousebound patients with myalgic encephalomyelitis and chronic fatigue syndrome. Chronic Illn (2016) 12(4):292–307. doi: 10.1177/1742395316644770 27127189 PMC5464362

[4] Montoya JG Holmes TH Anderson JN Maecker HT Rosenberg-Hasson Y Valencia IJ. Cytokine signature associated with disease severity in chronic fatigue syndrome patients. Proc Natl Acad Sci U.S.A. (2017) 114(34):E7150–E7158. doi: 10.1073/pnas.1710519114 PMC5576836 28760971

[5] Baklund IH Dammen T Moum TÅ Kristiansen W Duarte DS Castro-Marrero J. Evaluating routine blood tests according to clinical symptoms and diagnostic criteria in individuals with Myalgic Encephalomyelitis/Chronic Fatigue Syndrome. J Clin Med (2021) 10(14):3105. doi: 10.3390/jcm10143105 34300271 PMC8307418

[6] Germain A Ruppert D Levine SM Hanson MR. Metabolic profiling of a myalgic encephalomyelitis/chronic fatigue syndrome discovery cohort reveals disturbances in fatty acid and lipid metabolism. Mol Biosyst (2017) 13(2):371–9. doi: 10.1039/c6mb00600k PMC5365380 28059425

[7] Hornig M Montoya JG Klimas NG Levine S Felsenstein D Bateman L. Distinct plasma immune signatures in ME/CFS are present early in the course of illness. Sci Adv (2015) 1(1):e1400121. doi: 10.1126/sciadv.1400121 26079000 PMC4465185

[8] Deumer U-S Varesi A Floris V Savioli G Mantovani E López-Carrasco P. Myalgic encephalomyelitis/chronic fatigue syndrome (ME/CFS): An overview. J Clin Med (2021) 10(20):4786. doi: 10.3390/jcm10204786 34682909 PMC8538807

[9] Lidbury BA Kita B Lewis DP Hayward S Ludlow H Hedger MP. Activin B is a novel biomarker for chronic fatigue syndrome/myalgic encephalomyelitis (CFS/ME) diagnosis: a cross sectional study. J Transl Med (2017) 15(1):60. doi: 10.1186/s12967-017-1161-4 28302133 PMC5353946

[10] Nacul de Barros Kingdon Cliff Clark Mudie. Evidence of clinical pathology abnormalities in people with myalgic encephalomyelitis/chronic fatigue syndrome (ME/CFS) from an analytic cross-sectional study. Diagnostics (Basel) (2019) 9(2):41. doi: 10.3390/diagnostics9020041 30974900 PMC6627354

[11] Gravelsina S Vilmane A Svirskis S Rasa-Dzelzkaleja S Nora-Krukle Z Vecvagare K. Biomarkers in the diagnostic algorithm of myalgic encephalomyelitis/chronic fatigue syndrome. Front Immunol (2022) 13:928945. doi: 10.3389/fimmu.2022.928945 36300129 PMC9589447

[12] Kashi AA Davis RW Phair RD. The IDO metabolic trap hypothesis for the etiology of ME/CFS. Diagnostics (Basel) (2019) 9(3):82. doi: 10.3390/diagnostics9030082 31357483 PMC6787624

[13] Craddock TJA Fritsch P Rice MA del Rosario RM Miller DB Fletcher MA. A role for homeostatic drive in the perpetuation of complex chronic illness: Gulf war illness and chronic fatigue syndrome. PloS One [ (2014) 9(1):e84839. doi: 10.1371/journal.pone.0084839 24416298 PMC3885655

[14] Archard LC Bowles NE Behan PO Bell EJ Doyle D. Postviral fatigue syndrome: Persistence of Enterovirus RNA in muscle and elevated creatine kinase. J R Soc Med (1988) 81(6):326–9. doi: 10.1177/014107688808100608 PMC1291623 3404526

[15] O’Boyle S Nacul L Nacul FE Mudie K Kingdon CC Cliff JM. A natural history of disease framework for improving the prevention, management, and research on post-viral fatigue syndrome and other forms of Myalgic Encephalomyelitis/Chronic Fatigue Syndrome. Front Med (Lausanne) (2022) 8:688159. doi: 10.3389/fmed.2021.688159 35155455 PMC8835111

[16] Nacul L O’Boyle S Palla L Nacul FE Mudie K Kingdon CC. How myalgic encephalomyelitis/chronic fatigue syndrome (ME/CFS) progresses: The natural history of ME/CFS. Front Neurol (2020) 11:826. doi: 10.3389/fneur.2020.00826 32849252 PMC7431524

[17] Brown MM Bell DS Jason LA Christos C Bell DE. Understanding long-term outcomes of chronic fatigue syndrome: Long-term outcomes of CFS. J Clin Psychol (2012) 68(9):1028–35. doi: 10.1002/jclp.21880 PMC3940158 22753044

[18] Krabbe SH Groven KS Schrøder Bjorbækmo W Sveen U Mengshoel AM. The fragile process of Homecoming - Young women in recovery from severe ME/CFS. Int J Qual Stud Health Well-being (2023) 18(1):2146244. doi: 10.1080/17482631.2022.2146244 PMC9661980 36367977

[19] Pheley AM Melby D Schenck C Mandel J Peterson PK. Can we predict recovery in chronic fatigue syndrome? Minn. Med. (1999) 82(11):52–6. 10589213

[20] Sotzny F Blanco J Capelli E Castro-Marrero J Steiner S Murovska M. Myalgic Encephalomyelitis/Chronic Fatigue Syndrome – Evidence for an autoimmune disease. Autoimmun Rev (2018) 17(6):601–9. doi: 10.1016/j.autrev.2018.01.009 29635081

[21] Strand EB Nacul L Mengshoel AM Helland IB Grabowski P Krumina A. Myalgic encephalomyelitis/chronic fatigue Syndrome (ME/CFS): Investigating care practices pointed out to disparities in diagnosis and treatment across European Union. PloS One (2019) 14(12):e0225995. doi: 10.1371/journal.pone.0225995 31805176 PMC6894853

[22] Waters FG McDonald GJ Banks S Waters RA. Myalgic Encephalomyelitis (ME) outbreaks can be modelled as an infectious disease: a mathematical reconsideration of the Royal Free Epidemic of 1955. Fatigue (2020) 8(2):70–83. doi: 10.1080/21641846.2020.1793058

[23] Poenaru S Abdallah SJ Corrales-Medina V Cowan J. COVID-19 and post-infectious myalgic encephalomyelitis/chronic fatigue syndrome: a narrative review. Ther Adv Infect Dis (2021) 8:204993612110093. doi: 10.1177/20499361211009385 PMC8060761 33959278

[24] Kedor C Freitag H Meyer-Arndt L Wittke K Hanitsch LG Zoller T. A prospective observational study of post-COVID-19 chronic fatigue syndrome following the first pandemic wave in Germany and biomarkers associated with symptom severity. Nat Commun (2022) 13(1):5104. doi: 10.1038/s41467-022-32507-6 PMC9426365 36042189

[25] Araja D Berkis U Murovska M. COVID-19 pandemic-revealed consistencies and inconsistencies in healthcare: A medical and organizational view. Healthcare (Basel) (2022) 10(6):1018. doi: 10.3390/healthcare10061018 35742069 PMC9223168

[26] Esfandyarpour R Kashi A Nemat-Gorgani M Wilhelmy J Davis RW. A nanoelectronics-blood-based diagnostic biomarker for myalgic encephalomyelitis/chronic fatigue syndrome (ME/CFS). Proc Natl Acad Sci U.S.A. (2019) 116(21):10250–7. doi: 10.1073/pnas.1901274116 PMC6535016 31036648

[27] Missailidis D Sanislav O Allan CY Annesley SJ Fisher PR. Cell-based blood biomarkers for myalgic encephalomyelitis/chronic fatigue syndrome. Int J Mol Sci (2020) 21(3):1142. doi: 10.3390/ijms21031142 32046336 PMC7037777

[28] Sotzny F Filgueiras IS Kedor C Freitag H Wittke K Bauer S. Dysregulated autoantibodies targeting vaso- and immunoregulatory receptors in Post COVID Syndrome correlate with symptom severity. Front Immunol (2022) 13:981532. doi: 10.3389/fimmu.2022.981532 36238301 PMC9552223

[29] Fukuda K. The chronic fatigue syndrome: A comprehensive approach to its definition and study. Ann Intern Med (1994) 121(12):953. doi: 10.7326/0003-4819-121-12-199412150-00009 7978722

[30] Center for Disease Control. International research case definition (1994). Available at: https://www.cdc.gov/me-cfs/healthcare-providers/case-definitions-criteria.html.

[31] Carruthers BM van de Sande MI De Meirleir KL Klimas NG Broderick G Mitchell T. Myalgic encephalomyelitis: International consensus criteria. J Intern Med (2011) 270(4):327–38. doi: 10.1111/j.1365-2796.2011.02428.x PMC3427890 21777306

[32] Soldatos CR Dikeos DG Paparrigopoulos TJ. Athens Insomnia Scale: validation of an instrument based on ICD-10 criteria. J Psychosom Res (2000) 48(6):555–60. doi: 10.1016/s0022-3999(00)00095-7 11033374

[33] Minnock P Ringnér A Bresnihan B Veale D FitzGerald O McKee G. Perceptions of the cause, impact and management of persistent fatigue in patients with rheumatoid arthritis following tumour necrosing factor inhibition therapy: Patients’ perceptions of fatigue in rheumatoid arthritis. Musculoskeletal Care [Internet] (2017) 15(1):23–35. doi: 10.1002/msc.1136 26871999

[34] JASP 0.18 (JASP Team. JASP (Version 0.18). (2023). University of Amsterdam, Amsterdam, The Netherlands. https://jasp-stats.org/.

[35] Sullivan GM Feinn R. Using effect size—or why the P value is not enough. J Grad Med Educ (2012) 4(3):279–82. doi: 10.4300/jgme-d-12-00156.1 PMC3444174 23997866

[36] Serdar CC Cihan M Yücel D Serdar MA. Sample size, power and effect size revisited: simplified and practical approaches in pre-clinical, clinical and laboratory studies. Biochem Med (Zagreb) (2021) 31(1):27–53. doi: 10.11613/bm.2021.010502 PMC7745163 33380887

[37] Witten IH Frank E Hall M Pal C. Data mining: Practical machine learning tools and techniques. 4th ed. Oxford, England: Morgan Kaufmann (2011).

[38] Kim J-O Mueller CW. Factor analysis: Statistical methods and practical issues. Thousand Oaks, CA: SAGE Publications (1979).

[39] Jolliffe IT. Principal Component Analysis. 2nd ed. New York, NY: Springer (2002).

[40] Komaroff AL. Inflammation correlates with symptoms in chronic fatigue syndrome. Proc Natl Acad Sci U.S.A. (2017) 114(34):8914–6. doi: 10.1073/pnas.1712475114 PMC5576849 28811366

[41] Genuer R Poggi J-M. Random forests with R. 1st ed. Cham, Switzerland: Springer Nature (2020).

[42] Gravelsina S Nora-Krukle Z Vilmane A Svirskis S Vecvagare K Krumina A. Potential of activin B as a clinical biomarker in myalgic encephalomyelitis/chronic fatigue syndrome (ME/CFS). Biomolecules (2021) 11(8):1189. doi: 10.3390/biom11081189 34439855 PMC8394088

[43] Guenther S Loebel M Mooslechner AA Knops M Hanitsch LG Grabowski P. Frequent IgG subclass and mannose binding lectin deficiency in patients with chronic fatigue syndrome. Hum Immunol (2015) 76(10):729–35. doi: 10.1016/j.humimm.2015.09.028 26429318

[44] Xu J Lodge T Kingdon C Strong JWL Maclennan J Lacerda E. Developing a blood cell-based diagnostic test for myalgic encephalomyelitis/chronic fatigue syndrome using peripheral blood mononuclear cells. Adv Sci (Weinh) (2023) 10(30):2302146. doi: 10.1002/advs.202302146 PMC10602530 37653608

[45] Lidbury BA Kita B Richardson AM Lewis DP Privitera E Hayward S. Rethinking ME/CFS diagnostic reference intervals via machine learning, and the utility of activin B for defining symptom severity. Diagnostics (Basel) (2019) 9(3):79. doi: 10.3390/diagnostics9030079 31331036 PMC6787626

[46] Jäkel B Kedor C Grabowski P Wittke K Thiel S Scherbakov N. Hand grip strength andfatigability: correlation with clinical parameters and diagnostic suitability in ME/CFS. J Transl Med (2021) 19(1):159. doi: 10.1186/s12967-021-02774-w 33874961 PMC8056497

